# Online Behaviours during the COVID-19 Pandemic and Their Associations with Psychological Factors: An International Exploratory Study

**DOI:** 10.3390/ijerph19148823

**Published:** 2022-07-20

**Authors:** Julius Burkauskas, Naomi A. Fineberg, Konstantinos Ioannidis, Samuel R. Chamberlain, Henrietta Bowden-Jones, Inga Griskova-Bulanova, Aiste Pranckeviciene, Artemisa R. Dores, Irene P. Carvalho, Fernando Barbosa, Pierluigi Simonato, Ilaria De Luca, Rosin Mooney, Maria Ángeles Gómez-Martínez, Zsolt Demetrovics, Krisztina Edina Ábel, Attila Szabo, Hironobu Fujiwara, Mami Shibata, Alejandra R. Melero-Ventola, Eva M. Arroyo-Anlló, Ricardo M. Santos-Labrador, Kei Kobayashi, Francesco Di Carlo, Cristina Monteiro, Giovanni Martinotti, Ornella Corazza

**Affiliations:** 1 Laboratory of Behavioral Medicine, Neuroscience Institute, Lithuanian University of Health Sciences, 00135 Palanga, Lithuania; aiste.pranckeviciene@lsmuni.lt; 2 Department of Clinical, Pharmaceutical and Biological Sciences, School of Life and Medical Sciences, University of Hertfordshire, Hatfield AL10 9EU, UK; naomi.fineberg@btinternet.com (N.A.F.); pierluigi.simonato@gmail.com (P.S.); ilaria-deluca@hotmail.it (I.D.L.); giovanni.martinotti@gmail.com (G.M.); o.corazza@herts.ac.uk (O.C.); 3 Department of Psychiatry, University of Cambridge, Cambridge CB2 1TN, UK; konstantinos.ioannidis@cpft.nhs.uk (K.I.); hb584@cam.ac.uk (H.B.-J.); 4 Department of Psychiatry, Faculty of Medicine, University of Southampton, Southampton SO17 1BJ, UK; 5 Southern Health NHS Foundation Trust, Southampton SO40 2RZ, UK; 6 Department of Neurobiology and Biophysics, Institute of Biosciences, Vilnius University, 03225 Vilnius, Lithuania; inga.griskova-bulanova@gf.vu.lt; 7 School of Health, Polytechnic of Porto, 4200-072 Porto, Portugal; artemisa@ess.ipp.pt; 8 Laboratory of Neuropsychophysiology, Faculty of Psychology and Education Sciences, University of Porto, 4200-135 Porto, Portugal; fernandobarbosa@me.com; 9 Clinical Neurosciences and Mental Health Department, Faculty of Medicine, University of Porto, 4200-450 Porto, Portugal; irenec@med.up.pt; 10 CINTESIS@RISE, Faculty of Medicine, University of Porto, 4200-450 Porto, Portugal; 11 Medical Sciences Division, Department of Psychiatry, University of Oxford, Oxford OX1 3TG, UK; r.mooney@herts.ac.uk; 12 Department of Psychology, Pontifical University of Salamanca, 37002 Salamanca, Spain; magomezma@upsa.es (M.Á.G.-M.); amelero@cop.es (A.R.M.-V.); 13 Centre of Excellence in Responsible Gaming, University of Gibraltar, Gibraltar GX11 1AA, Gibraltar; zsolt.demetrovics@unigib.edu.gi; 14 Institute of Psychology, ELTE Eötvös Loránd University, 1117 Budapest, Hungary; abel.krisztina@ppk.elte.hu (K.E.Á.); szabo.attila@ppk.elte.hu (A.S.); 15 Institute of Health Promotion and Sport Sciences, ELTE Eötvös Loránd University, 1117 Budapest, Hungary; 16 Department of Neuropsychiatry, Graduate School of Medicine, University of Kyoto, Kyoto 606-8501, Japan; hirofuji@kuhp.kyoto-u.ac.jp (H.F.); mami_sh@kuhp.kyoto-u.ac.jp (M.S.); kkbys@kuhp.kyoto-u.ac.jp (K.K.); 17 Artificial Intelligence Ethics and Society Team, RIKEN Center for Advanced Intelligence Project, Saitama 103-0027, Japan; 18 General Research Division, Research Center on Ethical, Legal and Social Issues, Osaka University, Suita, Osaka 565-0871, Japan; 19 Department of Psychobiology, Neuroscience Institute of Castilla-León, University of Salamanca, 37002 Salamanca, Spain; anlloa@usal.es; 20 Department of Physical Education, University Teacher’s College ‘Fray Luis de León’, 47010 Valladolid, Spain; ricardo.santos@frayluis.com; 21 Department of Neuroscience, Imaging and Clinical Science “G. d’Annunzio” University of Chieti-Pescara, 66100 Chieti, Italy; francesco.dic@hotmail.it; 22 Department of Psychometrics, Institute of Psychology, Federal University of Rio de Janeiro, Rio de Janeiro 21941-901, Brazil; monteiro.cristina@yahoo.com.br

**Keywords:** problematic usage of the Internet, appearance anxiety, COVID-19 pandemic, mental illness, self-compassion

## Abstract

This cross-sectional study aimed to explore specific online behaviours and their association with a range of underlying psychological and other behavioural factors during the COVID-19 pandemic. Eight countries (Italy, Spain, the United Kingdom, Lithuania, Portugal, Japan, Hungary, and Brazil) participated in an international investigation involving 2223 participants (*M* = 33 years old; *SD* = 11), 70% of whom were females. Participants were surveyed for specific type of Internet use severity, appearance anxiety, self-compassion, and image and use of performance-enhancing drugs (IPEDs). Results were compared cross-culturally. The mean time spent online was 5 h (*SD* = ±3) of daily browsing during the pandemic. The most commonly performed activities included social networking, streaming, and general surfing. A strong association between these online behaviours and appearance anxiety, self-compassion, and IPEDs use was found after adjustment for possible confounders, with higher scores being associated with specific online activities. Significant cross-cultural differences also emerged in terms of the amount of time spent online during the initial stages of the COVID-19 pandemic.

## 1. Introduction

Problematic usage of the internet (PUI) encompasses a variety of problematic behaviours, including excessive general internet surfing, video gaming, pornography watching, shopping online, gambling online, social networking, cyberchondria, and digital hoarding [1]. In 2020, increased involvement in PUI was suggested across the globe, possibly due to the emergence of COVID-19 and the measures adopted to combat the spread of the virus, e.g., mandatory lockdown and mandatory quarantine [2,3,4]. However, little is known about any potential cross-cultural differences, including the amount of time spent online and whether this could be related to an increased PUI in the respective countries [3].

For example, a cross-sectional study conducted in China during COVID-19 involving 6416 individuals (*M* = 28; *SD* = 9 years old) and collecting self-reported behavioural change highlighted that 46.8% of the participants reported an increased internet dependence, with 16.6% of them showing a prolonged duration of internet use and 4.3% manifesting more severe dependence, measured by the internet addiction test (IAT) [5]. Another cross-sectional COVID-19 survey (*N* = 51,246) performed in Japan’s general population (*M* = 47; *SD* = 12 years old) showed probable PUI increase to be 7.8% overall, and 17.0% among younger people, higher than reported before the pandemic (3.2–3.7%) as measured using the Compulsive Internet Use Scale [6]. It should be noted that the thresholds used in such work are suggestive only since thresholds differ across countries using this instrument, and the thresholds used were not validated [7].

Research on 4734 adults in Indonesia (*M* = 32 *SD* = 8 years old) is noteworthy since it showed that, despite a probable increase in PUI, physical distancing alone was not associated with an increased risk of internet addiction, as measured by the Indonesian version of the Internet Addiction Diagnostic Questionnaire [8]. Thus, psychological, behavioural, social, economic, and other possible factors contributing to PUI in the context of the COVID-19 pandemic need to be investigated.

### 1.1. PUI and Underlying Psychological and Behavioural Factors

A previous investigation by our group among the general population identified a surge of problematic exercising, and appearance anxiety, during the COVID-19 lockdown period [9]. At that time, individuals were confined at home, gyms were closed, and a wide range of social restrictive measures were implemented by governments to safeguard public health. In order to cope with such a radical change in their lifestyles, and moved by concern about their self-image, almost one third of the respondents reported an unsupervised use of image and performance-enhancing drugs (IPEDs) to their boost image and performance and significant appearance anxiety levels [9,10]. Interestingly, appearance anxiety was previously linked to PUI by other authors [11], particularly social media use [12]. Body dissatisfaction has been correlated with PUI in both males’ and females’ recent meta-analysis (*N* = 32,295 participants) [13], and putative mechanisms for specific effects for the consumption of image-based content and other online applications (e.g., fitness tracking Apps, calorie counting Apps, and online dating) on appearance concerns (e.g., drive for thinness, body dissatisfaction, and excessive exercise) have been discussed [14]. Finally, there appears to be a link between PUI and specific over-exercising subgroups with appearance concerns (e.g., populations at risk of developing eating disorders) in which PUI mediates the relationship between obsessionality/sensation seeking and appearance-concern behaviour [15].

On the other hand, not only risk factors of PUI but also factors that might help to prevent unhealthy internet use need to be investigated. Positive coping and the phenomena of self-compassion especially drew researchers’ attention in the context of the COVID-19 pandemic [16]. Self-compassion was found to be associated with various types of mental distress as well as positive measures of quality of life and life satisfaction and is recognised as a measure of positive coping towards one’s inadequacies and life challenges [17]. For example, in a recent study of 617 Turkish adults (*M* age = 30.44, *SD* = 11.45 years old), self-compassion indirectly affected psychological well-being through mediating variables such as decreased psychological distress and increased resilience [18,19]. Self-compassion can be conceptualised as a coping and emotional regulation strategy that helps to maintain a healthy relationship with oneself in times and situations of suffering, failures, or general life difficulties [20]. A meta-analysis by Zessin et al. (*N* = 16,416) indicated a moderate relationship and a causal effect between self-compassion and cognitive and psychological well-being [19]. Although the relationship between self-compassion and PUI is not well researched, initial findings support the mediating role of self-compassion in internet addiction [21].

### 1.2. Socio-Demographic, Mental Health, and Other Behavioural Factors Contributing to PUI

Recent evidence has shown that besides well known socio-demographic (age, gender) and behavioural (e.g., time spent online) factors [1], general internet addiction scores are associated with worsened mental health, namely, anxiety and depression symptoms [22,23,24,25,26,27]. Additionally, there may be associations between aspects of PUI and various substance addictions. For example, a study conducted during the COVID-19 pandemic with a sample of 13,525 Bangladeshi participants (*M* = 24 *SD* = 8 years old) revealed associations between PUI, decreased physical exercise, and nicotine dependence (or tobacco use disorder) [28]. In addition, an Italian study with 1519 participants (*M* =29; *SD* =11 years old) found positive associations between the negative psychological consequences of COVID-19, alcohol use problems, and problematic social media usage [23]. Thus, the factors summarised here should be considered in the models explaining online behaviours during the COVID-19 pandemic.

Overall, the aforementioned findings suggest that PUI can be found amongst a cluster of potentially harmful behaviours or may represent coping attempts to alleviate mental distress and compensate for the lack of stimulation during the COVID-19 pandemic [29,30,31,32,33,34,35,36,37,38,39]. Prolonged exposure to screen time, and online information and advertisements during the confinement period, might have also had an impact on people’s mood, image, performance, physical exercise, and IPED consumption. Particularly, the time spent on social media (e.g., Facebook, Instagram) may not only have supported feelings of social connectedness when face-to-face contact was impossible [30] but also exposed users to body image contents, leading to maladaptive internet use for some vulnerable individuals. Within this context, our study aimed at:

(1) Exploring cross-national differences in time spent online and specific online behaviours during the start of the COVID-19 pandemic.
(2) Investigating associations between specific online behaviours during the start of the COVID-19 pandemic and psychological (appearance anxiety, self-compassion), and behavioural (exercising, use of IPEDs) factors while controlling for possible confounders, including demographic factors (age, gender), history of self-reported mental disorders (including self-reported anxiety, depression, and addictions), and cultural effects that may facilitate or else prevent PUI.

## 2. Materials and Methods

### 2.1. Procedure

The study was implemented in eight countries: Italy, Spain, United Kingdom, Lithuania, Portugal, Japan, Hungary and Brazil. Countries were invited to participate in the study with the support of a network of scientific collaborators, participating in the networks of COST Action CA 16207 (European Network for Problematic Usage of the Internet). A snowballing technique was used to recruit a convenience sample of individuals in the general public who use the internet. Researchers initially employed first-hand networks of COST Action CA 16207 to spread the study link, which was also shared through various social media platforms, including Facebook, Twitter, Instagram, and LinkedIn, to achieve an adequate study sample size.

Data collection was implemented using the web-based survey platform Qualtrics (Qualtrics, Provo, UT, 2020). Formal online consent was provided for each participant to tick before starting the survey. The survey provided information about the study (i.e., the study objectives and assurance of anonymity and confidentiality) and the various instruments. All the instruments were translated and back-translated from English into the different languages (Italian, Spanish, Japanese, Portuguese from Portugal and Brazil, Hungarian, and Lithuanian). No compensation was given upon completion, and the engagement rate was not monitored.

The survey was made available from April to June 2020 in all respective countries, except Brazil, where the study was conducted from May to June 2020. The timing of the study coincided with the initial lockdown period in Portugal, the UK, Italy, Spain, Hungary, Japan, Lithuania, and Brazil.

The data were stored in a secure platform at the University of Hertfordshire. Conventional data from this study sample (including IPEDs use/body image/exercise habits) were reported previously [9,40].

### 2.2. Ethics

The study received approval from the Human Sciences Ethics Committee at the University of Hertfordshire (HSK/SF/UH/00104), and from the Ethics Committees of each participating country, conforming to the principles outlined in the Declaration of Helsinki and with the European General Data Protection Regulation.

### 2.3. Participants

The median of the scale completion time was 19 (15–26) min. In sum, 2913 participants entered the study; however, 23 % (*n* = 690) either did not finish the study or provided blank sections in the survey; thus, their data were removed from the sample, leaving a final sample of 2223 individuals in total, from Italy (*n* = 621; 27.9%); Spain (*n* = 257; 11.6%); United Kingdom (*n* = 126; 5.7%); Lithuania (*n* = 221; 9.9%); Portugal (*n* = 172; 7.7%); Japan (*n* = 68; 3.1%); Hungary (*n* = 47; 2.1 %); and Brazil (*n* = 711; 32.0%).

The socio-demographic characteristics of study participants are displayed in Table 1. The mean age of study participants was 33 years old (*SD* =11), and the majority were females (*n* = 1557; 70%). Most of the participants were either employed (*n* = 1037; 46.7%) or actively studying (*n* = 709; 31.9%).

### 2.4. Instruments

Three major psychometric scales were analysed in the current work: the 12-item Internet Activities Scale from the Internet Severity and Activities Addiction Questionnaire (ISAAQ) [41], the Appearance Anxiety Inventory (AAI) [42], and the Self-Compassion Scale (SCS—short form) [43]. In addition to basic information on age and gender, additional questions were also included in the survey. The authors developed these to assess the presence of mental illness, including the level of physical activity and IPEDs consumption (please see the Supplementary Material).

A modified version of the 12-item Internet Activities Scale from ISAAQ [41] was used to assess self-reported online time spent on non-work or study-related activities using a Likert scale from 0 to 5. Higher scores represent more time spent engaging in a specific activity. The list of activities included general unstructured internet surfing, massively multiplayer online role-playing games (MMORPGs) and time wasters (including games and applications for which the activity lacks a specific benefit), other internet gaming (any internet gaming that does not fall under the previous categories), online shopping, online gambling, social networking, sports, pornography, media streaming, digital hoarding (including excessive acquisition or failure in deleting digital materials), and cyberchondria (including excessive online searches and checking illness-related information). In our sample, the Cronbach’s alpha of this instrument was 0.66, ranging from 0.54 to 0.76 for the different countries.

The AAI [42] was used to measure individuals’ anxiety levels related to body image. The self-report instrument is comprised of 10 items rated on a Likert scale from zero (not at all) to four (all the time). The maximum total score is 40 points, which represents severe appearance anxiety. Scores over the cut-off score of 21 indicate severe anxiety about body image. In our sample, the Cronbach’s alpha of this tool was 0.87, ranging from 0.81 to 0.89 for the different countries.

The SCS—short form [43] was used to measure study participants’ perceived self-compassion. The short form of the questionnaire comprises 12 items rated on a five-point Likert scale. The total score of the questionnaire ranges from 12 to 60, with a higher score representing a greater perceived degree of self-compassion and a cut-off score ≤ 27 indicating low self-compassion. In our sample, the Cronbach’s alpha of the tool was 0.82, ranging from 0.78 to 0.88 for the different countries in this study.

### 2.5. Statistical Analysis

SPSS for Windows, version 22.0.0 (SPSS Inc., Chicago, IL, USA), was used for statistical analyses. We used the Krukal—Wallis analysis to compare the hours spent online in different countries, and the Dunn–Bonferroni method was used for post-hoc multiple comparisons because the results showed unequal variances (i.e., heteroskedasticity) across countries and the number of subjects per country was uneven. Pearson or Spearman’s correlation analyses were used to examine the relationships between AAI, SCS, and IPEDs use scores (yes/no), self-reported mental illness history (including self-reported anxiety [yes/no]; depression [yes/no]); and addictive behaviours, including smoking [yes/no]), together with demographic indicators (age, sex) and PUI risk and protective factors (the duration of internet use, physical activity time), all concerning specific online activities. Bonferroni corrections were adopted to reduce Type I error probability due to multiple comparisons.

As 12 different online activities were presumed to be associated with various psychological, demographic, and PUI risk factors, the *p*-value was divided by the number of dependent variables (05/12 = 0.0042). Thus, a *p* < 0.004 was required for a factor to later be considered in the multivariable analysis. The multivariable analysis was needed to determine whether appearance anxiety, self-compassion, IPEDs use, and activity level remained independently associated with online activities after adjustment for demographic, self-reported history of mental illness, PUI risk factors, and country (a specific region where the response of the survey was submitted, coded as a dummy variable). The multivariable regression models included only of those variables that had been significantly associated with their respective online activity after adjustment in the Pearson’s or Spearman’s correlation analyses.

## 3. Results

### 3.1. Baseline Characteristics

Across the entire study sample, the mean time spent online was 5.2 h (*SD* = 3.4) of daily browsing during the pandemic. The most commonly performed activities included social networking, streaming, and general surfing. The sample’s mean score for appearance anxiety was 16.83 (*SD* = 5.54) out of 40, ranging from 10 to 39, with approximately 21% (*n* = 461) of participants scoring over the cut-off score of 21, indicating severe anxiety towards one’s body image. The group’s mean score in the Self-Compassion Scale was 30.95 (*SD* = 6.04) out of 60, ranging from 12 to 48, with 22% (*n* = 489) of participants scoring less than the cut-off score of 27, indicating low self-compassion.

Descriptive statistics of study variables are provided in Table 1.

Most of the participants in the study were generally physically active and engaged in at least one sports activity (*n* = 1945; 87.5%). Among all the surveyed participants, 724 (32.6%) reported IPEDs use (detailed IPEDs use analysis is presented elsewhere [9]). A history of mental illness was self-reported by 716 participants (32.2%); 544 (76.0%) of those self-reported experiencing anxiety; 207 (28.9%) self-reported experiencing depression; and 531 (74.0%) self-reported an addiction (including smoking).

Differences among countries were observed for the duration of internet use, χ^2^ (7, 2215) = 81.611, *p* < 0.001 (effect size d = 0.37). The Dunn–Bonferronni method-based post-hoc analysis showed that the total daily duration of internet use among Spanish participants was considerably lower than with participants in Italy (effect size d = 0.50), Portugal (effect size d = 0.42), Brazil (effect size d = 0.26), and the UK (effect size d = 0.57) (*p* < 0.001). Significant differences among countries were observed in the prevalence of online behaviours. A detailed analysis on this and other characteristics is provided in Supplementary Tables S3–S5.

### 3.2. Correlational Analysis

Correlation analyses showed that age, gender, and duration of internet use were correlated with specific online behaviours. Appearance anxiety and self-compassion were correlated with almost all online activities, with the exception of online gambling and online sports activities, which were uncorrelated with self-compassion scores. Positive correlations were also observed between IPEDs use and online shopping (*r*(2221) = 0.12 *p* < 0.001), gambling (*r*(2221) = 0.08 *p <* 0.001), sports (*r*(2221) = 0.09 *p* < 0.001), pornography (*r*(2221) = 0.12 *p* < 0.001), streaming (*r*(2221) = 0.08 *p <* 0.001), digital hoarding (*r*(2221) = 0.07 *p <* = 0.001), and cyberchondria (*r*(2221) = 0.11 *p* < 0.001).

Engaging in any type of sporting activity was negatively associated with skills games and time wasters (*r*(2221) = −0.09 *p* < 0.001), while there was a positive correlation between physical activity and online sports (*r*(2221) = 0.14 *p* < 0.001). Self-reported anxiety correlated positively with general surfing (*r*(2221) = 10 *p* < 0.001), massively multiplayer online role-playing games (*r*(2221) = 0.12 *p* < 0.001), skills games and time wasters (*r*(2221) = 0.08 *p* < 0.001), internet gaming (*r*(2221) = 0.14 *p* < 0.001), online shopping (*r*(2221) = 0.07 *p* = 0.001), social networking (*r*(2221) = 0.13 *p* < 0.001), pornography (*r*(2221) = 0.07 *p* = 0.001), streaming (*r*(2221) = 0.13 *p* < 0.001), digital hoarding (*r*(2221) = 0.09 *p* < 0.001), and cyberchondria (*r*(2221) = 0.19 *p* < 0.001). Self-reported depression correlated positively with general surfing (*r*(2221) = 0.09 *p* < 0.001), internet gaming (*r*(2221) = 0.08 *p* < 0.001), pornography (*r*(2221) = 0.08 *p* < 0.001), streaming (*r*(2221) = 0.10 *p* < 0.001), digital hoarding (*r*(2221) = 0.08 *p* < 0.001), and cyberchondria (*r*(2221) = 0.09 *p* < 0.001). Self-reported addiction behaviours (including smoking) were associated with more than half of the online activities surveyed, including general surfing (*r*(2221) = 0.07 *p* = 0.001), multiplayer online role-playing games (*r*(2221) = 0.10 *p* < 0.001), skills games and time wasters (*r*(2221) = 0.13 *p* < 0.001), internet gaming (*r*(2221) = 0.08 *p* < 0.001), online gambling (*r*(2221) = 0.07 *p =* 0.001), pornography (*r*(2221) = 0.20 *p* < 0.001), and streaming (*r*(2221) = 0.07 *p* = 0.001). Correlation analyses are provided in Table 2.

### 3.3. Multivariable Modelling

In order to explore the specific factors associated with different online activities, we adjusted for the possible overriding effects of age, gender, country, and duration of internet use. After theseadjustments, appearance anxiety remained associated with general surfing (β = 0.134; *p* < 0.001), online shopping (β = 0.139; *p* < 0.001), social networking (β = 0.131; *p* < 0.001), pornography use (β = 0.100; *p* < 0.001), digital hoarding (β = 0.079; *p* = 0.001), and cyberchondria (β = 0.123; *p* <.001). Self-compassion scores were negatively associated with higher levels of skills games and time wasters (β = −0.059; *p* = 0.015), streaming (β = −0.055; *p* = 0.017), digital hoarding (β = −0.067; *p* = 0.006), and cyberchondria (β = −0.139; *p* < 0.001). IPEDs use was associated with online shopping (β = 0.083; *p* < 0.001), online gambling (β = 0.050; *p* = 0.023), pornography (β = 0.060; *p* < 0.001), streaming (β = 0.051; *p* = 0.010), and cyberchondria (β = 0.052; *p* = 0.014). Negative associations remained between engaging in any type of sporting activity and skills games and time wasters (β = −0.062; *p* = 0.004), while positive associations were observed between sporting activity and online sports (β = 0.170; *p* < 0.001). Several associations remained between self-reported addiction (including smoking) and massively multiplayer online role-playing games (β = 0.034; *p* = 0.037), skills games and time wasters (β = 0.065; *p* = 0.002), online gambling (β = 0.090; *p* < 0.001), and pornography (β = 0.086; *p* < 0.001). The multivariable model is provided in Table 3.

We used post-hoc power analysis to determine the effect size (as measured by Cohen’s f) of our findings. The effect sizes ranged from very small for online gambling (f^2^ = 0.03), online shopping (f^2^ = 0.07), internet gaming (f^2^ =.09), digital hoarding (f^2^ =.10), and skills games and time wasters (f^2^ =.10); small for cyberchondria (f^2^ =.11), massively multiplayer online role-playing games (f^2^ =.16), and general internet surfing (f^2^ =.18); moderate for streaming (f^2^ = 0.25) and social networking (f^2^ = 0.31); to large for pornography use (f^2^ = 0.65).

## 4. Discussion

This cross-sectional study aimed mainly to describe the specific online behaviours among the sample of eight countries while exploring potential associations between specific online behaviours and psychological, behavioural, and demographic factors. The results showed that appearance anxiety, self-compassion, IPEDs use, and engagement in any sporting activities were correlated to various forms of online activities.

### 4.1. Cross-National Differences in Time Spent Online and Specific Online Behaviours during the Start of the COVID-19 Pandemic

Previous studies reported increased internet use during the COVID-19 pandemic [2,3,4]. In the current study, participants reported spending on average 5 ± 3 h online, daily, after excluding time spent for work or study activities. Significant differences across countries, in terms of duration of internet use with a greater degree of general internet browsing behaviour, were observed for participants in the UK, Italy, and Portugal. This finding may reflect cultural differences in internet use. We propose that these countries might have been the ones experiencing the highest COVID-19 burden [44,45], and therefore more restrictions—including restricted travelling, schools, bars, clubs, gyms, concerts, etc.—than did the others. The greater confinement might have led individuals to spend more time online than respondents from other countries.

### 4.2. Appearance Anxiety, Exercise, Use of IPEDs

Appearance anxiety was found to be associated with the following online activities: general surfing, online shopping, online gambling, social networking, pornography use, digital hoarding, and cyberchondria. Individuals may use these activities to try to achieve and maintain a body image that corresponds to the valued ideas of beauty, sometimes with devastating effects [46]. Modern and Westernised societies attribute great importance to physical appearance. Although this phenomenon is not new, there is a higher and growing social pressure to achieve socially established beauty standards, mainly in modern and Westernised societies. This phenomenon may also have been boosted by the circumstances related with the COVID-19 pandemic. When the social ideals of beauty are unrealistic, they may result in dissatisfaction with one’s own body, appearance anxiety, and body image disorder [9,46]. Recent studies have suggested that active involvement in social networks can negatively influence body image and possibly be associated with body dissatisfaction and the use of IPEDs, among other effects [9,47]. On the other hand, studies have also found that internet use positively affects physical activity [48]. For example, in a study of Goodyear et al. (*N*  =  786; Mean age 45.1  ±  19.1 years old), social media positively facilitated self-management of physical activity and diet and contributed to increased health and well-being.

In this study, IPEDs use was associated with increased online shopping, pornography, streaming, and cyberchondria. Engaging in any type of live sporting activity was negatively associated with increased levels of skills games and time-wasters. However, being engaged in live sports activities was associated with increased levels of online sports. The increased preference for live sports activities could be generalised to online sports and promote the belief that the mastery and knowledge of live sporting activities can benefit online activity. The associations found are new, and no other studies explored general physical activity and its association with online behaviours from a cross-cultural perspective.

### 4.3. Self-Compassion

Higher self-compassion was negatively associated with increased skills games and time wasters, streaming, digital hoarding, and cyberchondria. Several previous studies showed negative associations between self-compassion and PUI [21,49,50]. However, our study is among the first to show associations between self-compassion and *specific* online behaviour. Our findings support the idea that self-compassion could be a positive coping recourse in adverse life circumstances. Previous research indicates that self-compassion could be boosted with relatively simple interventions, including online training [51]. Thus, investigating the self-compassion training effect as a possible intervention for PUI-related problems would be of high interest.

However, we have to acknowledge that the magnitude of the associations for appearance anxiety, self-compassion, IPEDs use, and general engagement in sporting activity ranged from being very small (e.g., for online gambling, digital hoarding, online shopping, skills games, time-wasters, and cyberchondria) to small/moderate (e.g., for general internet surfing, streaming, and social networking) and to large (e.g., for pornography use). These findings indicate a complexity of different online behaviours, as well as different factors that contribute to the development of particular online behaviours. Thus, more research is needed focusing on specific online behaviours rather than on PUI in general. Therefore, we further discuss the four most robust models explaining online activities during the initial COVID-19 period.

### 4.4. General Online Surfing

Our data showed that general internet surfing, consisting of any unstructured online activities, was associated with appearance anxiety. Specifically, we found that PUI correlated with AAI, which is in line with the recent meta-analysis that showed small–moderate correlations between PUI and body dissatisfaction in both men and women. In our study, appearance anxiety as well as PUI were self-reported. Therefore, stronger relationships between the proposed factors might be expected in clinically relevant online addictions. However, the idea that variables such as appearance anxiety may contribute to the general level of online activities creates a possibility for a better understanding of the formation of addictive online behaviours and, therefore, earlier targeted interventions.

### 4.5. Streaming

Online streaming, including music or video, on any platform, was associated with lower levels of self-compassion, more IPEDs use, younger age, and greater duration of internet use. A survey of 490 video streaming service users found that excessive use was associated with low self-control and self-esteem [52]. However, the motivation for online streaming should be included in the prediction models for better to explain this particular online behaviour [53].

### 4.6. Social-Networking

The association between social networking and appearance anxiety is consistent with the research literature in the field to date [54,55,56,57,58]. A large sample size study (*N* = 23,533) conducted within the Norwegian population confirmed the association between general anxiety and the addictive use of social media [59]. Furthermore, in a recent review by Ryding and Kuss (2020), the authors state that appearance-based comparisons were found to act as mediators between social networking and body image dissatisfaction [12]. This notion complements the works of other authors finding specific links between social anxiety and excessive social media use [56,57].

### 4.7. Pornography Use

Engagement in online pornography activity during the initial phases of the COVID-19 pandemic was associated with almost all variables examined in the current study. Specifically, online pornography use was associated with higher levels of appearance anxiety, IPEDs use, younger age, male gender, self-reported addictions (including smoking), and duration of internet use. Various studies have confirmed associations between pornography use frequency and younger age, male gender, and self-reported addiction [60,61,62,63,64]. In addition, these studies also find higher frequencies of pornography use to be associated with appearance anxiety. In their study, Tylka et al. (2015) [63] showed that men’s frequency of pornography use was positively associated with dissatisfaction with their bodies. According to our study, the use of IPEDs might also contribute to the frequency-of-pornography-use prediction models. Another consideration of why the observed association might be stronger is the special circumstances of the COVID-19 pandemic during which the study took place [64,65]. During the lockdown physical contact was limited, couples found themselves separated in different households, and dating was restricted due to health concerns. Those social structure changes may have impacted how people turned to online pornography use for their satisfaction of sexual needs, recreation, or anxiety management. Whether this possible increased turn to online pornography content is adaptive or maladaptive remains questionable, and studies with longitudinal design [66] might be able to identify, in the future, any existing effects or influences regarding this from the use of IPEDs.

## 5. Limitations

Despite these novel results, our study has some limitations. Due to the large sample size and statistical power of the analyses, the associations of a small effect would have been detected, but such associations may not be of practical relevance, i.e., such findings may pose no clinical significance for psychological or health interventions or ecological significance. Another limitation concerns the cross-sectional design that prevented any conclusions concerning causality in the significant associations. The study was advertised online as seeking to find out the impact of COVID-19 on individuals’ lifestyle behaviours. This type of advertisement might result in possible recruitment and responder bias (e.g., only those with access to the internet and mobile devices were able to access the survey). As the survey contained questions about PUI and related behaviours (e.g., pornography viewing), this must be taken into consideration, given that the nature of the variables may have resulted in the over- or under-reporting of these kinds of behaviours.

Our analysis also included a standard set of factors for each online behaviour model. However, several additional factors such as motivation, escapism tendencies [53], impulsivity [24,67,68], and compulsivity measures [1] should be introduced to better contextualise excessive online behaviours. Another set of variables that was not included in this analysis is exposure to COVID-19 (direct or indirect). Concerns over increased online gaming, gambling, and pornography use [4,32,35,69] during the COVID-19 pandemic were raised. However, it is unclear whether the pandemic resulted in an increase in other activities attributed to PUI such as online shopping, digital hoarding, sports, or cyberchondria. Negative consequences of increased time on the internet might be triggered by financial hardship [70], isolation [71], increased substance use, mental disorders, and other behavioural addictions [9,72,73]. These factors might also play a role in associations between specific online activities and appearance anxiety, self-compassion, and IPEDs use. While we controlled our findings for self-reported depression and anxiety, as well as for addiction scores, other factors such as financial burden or isolation were not explored, thus warranting further studies to investigate this possible relationship.

The large sample size enabled us to observe minor effects. Thus, replication is required for the findings presented. Appearance anxiety, self-compassion, IPEDs use, and general engagement in sporting activity were all associated with different online activities, and the significance of these associations remained strong even after controlling for possible confounders, suggesting several modelling approaches for a better understanding of the factors contributing to different types of online behaviours. Based on the effect sizes, the models relevant for further investigation should include general internet surfing, streaming, social networking, and pornography use.

## 6. Conclusions

In conclusion, our study showed the relationship between appearance anxiety, self-compassion, IPEDs use, and engagement in any sporting activity and various online activities. The strongest effects were observed for general internet surfing, streaming, social networking, and pornography use. Particularly, models including AAI, IPEDs use, age, gender, self-reported addiction, and duration of internet use factors were among the strongest predicting pornography use. Importantly, our study findings imply that not only well-known sociodemographic and mental distress factors are associated with engagement in online activities, but also appearance anxiety, self-compassion, IPEDs use, and general engagement in sporting activity are related to the various forms of online behaviour.

**Main contributions**

- Cross-national differences are present in time spent online and the intensity of various forms of online behaviours.
- We found that specific novel psychological and behavioural factors such as appearance anxiety, self-compassion, IPEDs use, and engagement in any sporting activity and various online activities might contribute to various forms of online behaviours.
- General internet surfing, streaming, social networking, and pornography-use-prediction models should include appearance anxiety, self-compassion, IPEDs use, age, gender, self-reported addiction, and duration of internet use.

## Figures and Tables

**Table 1:** Demographic characteristics, internet activities, Appearance Anxiety Inventory (AAI), and Self-Compassion Scale (SCS) scores.

	Total *n* = 2223
**Age, *M* ± *SD***	32.94 ± 11.26
**Sex (percent of the total sample), *n* (%)**	
Men	666 (30.0%)
Women	1557 (70.0%)
**Occupation, *n* (%)**	
Employed	1037 (46.68%)
Student	709 (31.9%)
Unemployed	172 (7.7%)
Retired	105 (4.7%)
Freelance/individual activity	200 (9.0%)
**Internet activities, *M* ± *SD***	
General surfing	3.36 ± 1.00
Massively multiplayer online role-playing games	1.54 ± 1.03
Skills games and time wasters	2.01 ± 1.20
Internet gaming	1.58 ± 0.98
Online shopping	2.36 ± 1.02
Online gambling	1.08 ± 0.39
Social networking	3.75 ± 1.06
Sports	2.27 ± 1.26
Pornography viewing	1.85 ± 1.11
Streaming	3.43 ± 1.19
Digital hoarding	2.06 ± 1.15
Cyberchondria	1.68 ± 0.96
**Activity (engaging in at least one sport), *n* (%)**	1945 (87.5%)
**Subjectively reported history of mental illness, *n* (%)**	716 (32.2%)
Anxiety	544 (76.0%)
Depression	207 (28.9%)
Addiction (including smoking)	531 (23.9%)
**Appearance Anxiety Inventory, *M* ± *SD***	16.83 ± 5.54
**Self-Compassion Scale, *M* ± *SD***	30.95 ± 6.04
**Image and performance-enhancing drugs used, *n* (%)**	724 (32.6%)
**Browsing online hours per day during periods of increased social/physical distance, *M* ± *SD***	5.17 ± 3.38

**Table 2:** Spearman correlation between self-reported internet activities and demographic, clinical, and psychological characteristics.

*N* = 2223	.	General Surfing	Massively Multiplayer Online Role- Playing Games	Skills Games & Time Wasters	Internet Gaming	Online Shopping	Online Gambling	Social Networking	Sports	Pornography	Streaming	Digital Hoarding	Cyberchondria
AAI	r(p)	0.254 (<0.001)	0.104 (<0.001)	0.094 (<0.001)	0.088 (<0.001)	0.173 (<0.001)	0.057 (0.007)	0.324 (<0.001)	0.083 (<0.001)	0.118 (<0.001)	0.218 (<0.001)	0.148 (<0.001)	0.205 (<0.001)
SCS	r(p)	−0.197 (<0.001)	−0.115 (<0.001)	−0.144 (<0.001)	−0.086 (<0.001)	−0.072 (0.001)	−0.024 (0.260)	−0.219 (<0.001)	−0.031 (0.138)	−0.139 (<0.001)	−0.200 (<0.001)	−0.114 (<0.001)	−0.188 (<0.001)
IPEDs	r(p)	0.025 (0.239)	0.049 (0.022)	−0.056 (0.009)	−0.003 (0.900)	0.116 (<0.001)	0.084 (<0.001)	0.042 (0.046)	0.088 (<0.001)	0.120 (<0.001)	0.078 (<0.001)	0.071 (0.001)	0.109 (<0.001)
Physical Activity	r(p)	−0.022 (0.292)	0.008 (0.716)	−0.094 (<0.001)	0.024 (0.255)	0.034 (0.113)	0.025 (0.235)	−0.003 (0.875)	0.142 (<0.001)	−0.007 (0.731)	0.031 (0.150)	0.060 (0.005)	0.012 (0.576)
Age	r(p)	−0.308 (<0.001)	−0.304 (<0.001)	−0.200 (<0.001)	−0.178 (<.001)	−0.027 (0.198)	−0.060 (0.005)	−0.387 (<0.001)	−0.148 (<0.001)	−0.239 (<0.001)	−0.378 (<0.001)	−0.074 (0.001)	−0.043 (0.041)
Gender	r(p)	−0.012 (0.557)	−0.210 (<0.001)	−0.109 (<0.001)	−0.179 (<0.001)	0.100 (<0.001)	−0.136 (<0.001)	0.140 (<0.001)	0.135 (<0.001)	−0.514 (<0.001)	−0.014 (0.502)	−0.027 (0.207)	0.055 (0.010)
Duration of internet use (h)	r(p)	0.256 (<0.001)	0.154 (<0.001)	0.124 (<0.001)	0.123 (<0.001)	0.073 (0.001)	0.052 (0.015)	0.391 (<0.001)	0.076 (<0.001)	0.185 (<0.001)	0.218 (<0.001)	0.097 (<0.001)	0.101 (<0.001)
Self-reported history of mental illness													
Anxiety	r(p)	0.102 (<0.001)	0.122 (<0.001)	0.076 (<0.001)	0.135 (<0.001)	0.073 (0.001)	0.011 (0.594)	0.129 (<0.001)	0.018 (0.393)	0.073 (0.001)	0.133 (<0.001)	0.094 (<0.001)	0.186 (<0.001)
Depression	r(p)	0.087 (<0.001)	0.050 (0.017)	0.044 (0.039)	0.081 (<0.001)	0.038 (0.073)	−0.018 (0.392)	0.054 (0.011)	−0.010 (0.634)	0.082 (<0.001)	0.097 (<0.001)	0.082 (<0.001)	0.093 (<0.001)
Addiction (including smoking)	r(p)	0.069 (0.001)	0.102 (<0.001)	0.126 (<0.001)	0.078 (<0.001)	−0.025 (0.237)	0.074 (0.001)	0.030 (0.155)	−0.002 (0.939)	0.202 (<0.001)	0.071 (0.001)	−0.003 (0.884)	0.003 (0.895)

Note: AAI—Appearance Anxiety Inventory; SCS—Self-Compassion Scale; and IPEDs—image and performance-enhancing drugs.

**Table 3:** Multivariable modelling, including self-reported internet activities and demographic, clinical, and psychological characteristics.

	General Surfing	Massively Multiplayer Online Role- Playing Games	Skills Games and Time Wasters	Internet Gaming	Online Shopping	Online Gambling	Social Networking	Sports	Pornography	Streaming	Digital Hoarding	Cyberchondria
AAI	0.134 (<0.001)	0.037 (0.121)	0.043 (0.078)	0.025 (0.307)	0.139 (<0.001)	-	0.131 (<0.001)	−0.008 (0.725)	0.100 (<0.001)	0.032 (0.166)	0.079 (0.001)	0.123 (<0.001)
SCS	−0.024 (0.318)	−0.024 (300)	−0.059 (0.015)	−0.020 (0.411)	0.018 (0.470)	-	−0.008 (0.711)	-	−0.029 (0.144)	−0.055 (0.017)	−0.067 (0.006)	−0.139 (<0.001)
IPEDs	-	-	-	-	0.083 (<0.001)	0.050 (0.023)	-	0.115 (<0.001)	0.060 (<0.001)	0.051 (0.010)	0.039 (0.063)	0.052 (0.014)
Physical Activity	-	-	−0.062 (0.004)	-	-	-	-	0.170 (<0.001)	-	-	-	-
Age	−0.214 (<0.001)	−0.211 (<0.001)	−0.142 (<0.001)	−0.109 (<0.001)	-	-	−0.247 (<0.001)	−0.166 (<0.001)	−0.153 (<0.001)	−0.262 (<0.001)	0.001 (0.976)	-
Gender	-	−0.229 (<0.001)	−0.112 (<0.001)	−0.185 (<0.001)	0.097 (<0.001)	−0.109 (<0.001)	0.129 (<0.001)	0.139 (<0.001)	−0.520 (<0.001)	-	-	-
Mental illness												
Anxiety	0.005 (0.824)	0.046 (0.045)	0.071 (0.003)	0.052 (0.030)	0.029 (0.223)	-	−0.010 (0.655)	-	−0.008 (0.667)	−0.025 (0.264)	−0.037 (0.122)	0.090 (<0.001)
Depression	0.024 (0.263)	-	-	0.028 (0.200)	-	-	-	-	0.025 (0.163)	0.013 (0.536)	0.023 (0.297)	−0.017 (0.452)
Addiction (including smoking)	0.018 (0.376)	0.043 (0.037)	0.065 (0.002)	0.060 (0.004)	-	0.090 (<0.001)	-	-	0.086 (<0.001)	0.028 (0.158)	-	-
Duration of internet use (h)	0.102 (<0.001)	0.083 (<0.001)	0.071 (0.001)	0.101 (<0.001)	0.037 (0.083)	-	0.225 (<0.001)	0.020 (0.343)	0.078 (<0.001)	0.095 (<0.001)	0.091 (<0.001)	0.052 (0.014)
R^2^	0.158	0.146	0.098	0.105	0.075	0.033	0.241	0.101	0.398	0.203	0.097	0.108
df	14; 2208	14; 2208	15; 2207	15; 2207	13; 2209	10; 2212	13; 2209	13; 2209	16; 2206	15; 2207	14; 2208	13; 2209
F	290.568	260.864	160.024	170.225	130.782	70.462	530.830	190.195	910.261	370.525	160.871	200.621
*p*	<0.001	<0.001	<0.001	<0.001	<0.001	<0.001	<0.001	<0.001	<0.001	<0.001	<0.001	<0.001

Note: All variables found to be statistically significant in adjusted correlation modelling (*p* < 0.00417) were included into multivariable linear regression models while controlling for country. AAI—Appearance Anxiety Inventory; SCS—Self-Compassion Scale; and IPEDs—image and performance-enhancing drugs.

## Data Availability

The datasets supporting the conclusions of this article are available on request to Prof. Ornella Corazza.

## Supplementary Materials

The following supporting information can be downloaded at: https://www.mdpi.com/article/10.3390/ijerph19148823/s1, Table S1: Exact questions asked in the survey about self-reported mental illness, addictions, physical activity and IPEDs use; Table S2: Cross-national differences in socio-demographic, psychological and behavioural characteristics; Table S3: Cross-national differences in time spent online and specific online behaviours during the start of the COVID-19 pandemic; Table S4: Mean scores of appearance anxiety self-compassion measures and time spent online across countries; Table S5: Cross cultural comparisons of time spent online, appearance anxiety and self-compassion measures; Figure S1: Appearance anxiety, self-compassion measures and time spent online in different countries.
