# Supplementary material for: Online Behaviours during the COVID-19 Pandemic and Their Associations with Psychological Factors: An International Exploratory Study
Source: Int J Environ Res Public Health. 2022 Jul 20;19(14):8823. doi: 10.3390/ijerph19148823 (PMC9323465; doi:10.3390/ijerph19148823)
Supplement: Supplementary file 1 [file ijerph-19-08823-s001.zip › ijerph-1769475-supplementary.pdf]

**Table S1.** Exact questions asked in the survey about self-reported mental illness, addictions, physical activity and IPEDs use.

| |
|---|
| **Self-reported mental illness** |
| Do you suffer from any mental illness? [yes prior to physical distancing/yes since physical distancing/no] |
| If yes, please select which apply<br>o Anxiety<br>o Depression<br>o Other mood disorder(s)<br>o Psychotic disease<br>o Eating disorder<br>o Personality disorder<br>o Other |
| Has this period of physical distancing worsened your mental illness? [yes/no] |
| Have you ever struggled with addiction? (alcohol, drugs, gambling, etc.) [yes prior to physical distancing/yes since physical distancing/no] |
| If yes, has this physical distancing worsened your addiction problem? [yes/no] |
| **Alcohol use and smoking** |
| Do you smoke or vape? [yes/no] |
| Do you smoke/vape more than usual during physical distancing? [yes/no] |
| Before physical distancing, how often did you have a drink containing alcohol?<br>o Never<br>o Monthly or less<br>o 2-4 times a month<br>o 2-3 times a week<br>o 4 or more times a week |
| How many units of alcohol did you drink on a typical day before physical distancing? (1 unit = a small glass of 13% wine, a single shot of 40% spirit, a pint of beer)<br>o 1 or 2<br>o 3 or 4<br>o 5 or 6<br>o 7 to 9<br>o 10 or more |
| Since physical distancing, how often do you have a drink containing alcohol?<br>o Never<br>o Monthly or less<br>o 2-4 times a month<br>o 2-3 times a week<br>o 4 or more times a week |
| Since physical distancing, how many units of alcohol do you drink on an average day? (1 unit = a small glass of 13% wine, a single shot of 40% spirit, a pint of beer)<br>o 1 or 2<br>o 3 or 4 |

| |
|---|
| o 5 or 6<br>o 7 to 9<br>o 10 or more |
| **Physical activity** |
| What kind of sport/fitness activity did you engage in before physical distancing? |
| Are you a professional athlete? [yes/no] |
| Do you compete in your sport? (if yes, please state the professional body/hobby) [yes/no] |
| How many minutes per week did you spend doing moderate activity or training before physical distancing? |
| For how many months had you been training at this intensity? |
| How many minutes per week do you tend to spend doing moderate activity or training during this period of physical distancing? |
| Have you had a significant change in your fitness routine during this period? [yes/no] |
| Have you experienced any injuries during this time due to exercise? [yes/no] |
| To what extent does fitness help you cope with the current situation? [0-10] |
| **IPEDs use** |
| Have you ever taken supplements/products to help you reach your fitness goals/enhance your physical appearance? [yes/no] |
| If yes, what were they? |
| Where did you purchase them from during physical distancing? |
| Have you ever asked medical supervision or professional advice on the intake of these products? [yes/no] |

**Table S2.** Cross-national differences in socio-demographic, psychological and behavioural characteristics.

| | UK n = 126 | Italy n = 621 | Spain n = 257 | Hungary n = 47 | Portugal n = 172 | Japan n = 68 | Brazil n = 711 | Lithuania n = 221 | *p* |
|---|---|---|---|---|---|---|---|---|---|
| Age | 36.97±11.16 | 30.53±9.42 | 37.98±13.49 | 41.34±11.61 | 34.05±10.28 | 39.18±10.60 | 29.97±10.93 | 36.52±9.64 | **<0.001** |
| **Sex** | | | | | | | | | **<0.001** |
| Men | 45(35.7%) | 190(30.6%) | 79(30.7%) | 15(31.9%) | 29(16.9%) | 35(51.5%) | 245(34.5%) | 28(12.7%) | |
| Women | 81(64.3%) | 431(69.4%) | 178(69.3%) | 32(68.1%) | 143(83.1%) | 33(48.5%) | 466(65.5%) | 193(87.3%) | |
| **Occupation** | | | | | | | | | **<0.001** |
| Employed | 77(61.1%) | 303(48.8%) | 132(51.4%) | 32(68.1%) | 70(40.7%) | 59(86.8%) | 188(26.4%) | 176(79.6%) | |
| Student | 16(12.7%) | 165(26.5%) | 51(19.8%) | 2(4.3%) | 46(26.7%) | 6(8.8%) | 400(56.3%) | 23(10.4%) | |
| Unemployed | 4(3.2%) | 71(11.4%) | 27(10.5%) | 3(6.4%) | 13(7.6%) | 1(1.5%) | 48(6.8%) | 5(2.3%) | |
| Retired | 2(1.6%) | 80(12.9%) | 8(3.1%) | 2(4.3%) | 1(0.6%) | 0(0.0%) | 12(1.7%) | 0(0.0%) | |
| Freelance / individual activity | 27(21.4%) | 2(0.3%) | 39(15.2%) | 8(17.0%) | 42(24.4%) | 2(2.9%) | 63(8.9%) | 17(7.7%) | |
| **Activity (Engaging in at least one sport)** | 123(97.6%) | 484(77.9%) | 212(82.5%) | 45(95.7%) | 143(83.1%) | 56(82.4%) | 711(100%) | 171(77.4%) | **<0.001** |
| **Mental Illness** | 31(24.6%) | 137(22.1%) | 52(20.2%) | 5(10.6%) | 37(21.5%) | 8(11.8%) | 426(59.9%) | 20(9.0%) | **<0.001** |
| Anxiety | 25(80.6%) | 67(48.9%) | 40(76.9%) | 3(60.0%) | 24(64.9%) | 3(37.5%) | 370(86.9%) | 12(60.0%) | **<0.001** |
| Depression | 18(58.1%) | 23(16.8%) | 10(19.2%) | 2(40.0%) | 7(18.9%) | 1(12.5%) | 136(31.9%) | 10(50.0%) | **<0.001** |
| Addiction+ smoking | 26(20.6%) | 233(37.5%) | 63(24.5%) | 10(21.3%) | 25(14.5%) | 6(8.8%) | 122(17.2%) | 46(20.8%) | **<0.001** |
| **Appearance anxiety inventory** | 16.09±5.47 | 16.28±5.25 | 14.78±4.08 | 16.62±4.94 | 16.37±5.22 | 16.01±4.81 | 18.55±6.24 | 16.30±4.55 | **<0.001** |
| **Self-compassion scale** | 29.79±6.52 | 29.79±5.18 | 32.43±5.65 | 33.43±7.02 | 33.10±5.49 | 32.97±5.58 | 29.99±6.44 | 33.45±5.64 | **<0.001** |
| **Image and performance enhancing drugs use** | 36(28.6%) | 132(21.3%) | 41(16.0%) | 27(57.4%) | 50(29.1%) | 31(45.6%) | 329(46.3%) | 78(35.3%) | **<0.001** |

**Table S3.** Cross-national differences in time spent online and specific online behaviours during the start of the COVID-19 pandemic.

| | UK n = 126 1 | Italy n = 621 2 | Spain n = 257 3 | Hungary n = 47 4 | Portugal n = 172 5 | Japan n = 68 6 | Brazil n = 711 7 | Lithuania n = 221 8 | p | p* |
|---|---|---|---|---|---|---|---|---|---|---|
| **Internet activities** | | | | | | | | | | |
| General Surfing | 3.21±1.06 | 3.53±0.94 | 3.24±0.99 | 2.81±1.10 | 2.93±0.90 | 3.62±0.98 | 3.49±1.00 | 3.05±1.02 | **<0.001** | 2:3(0.001); 2:4(<0.001); 2:5(<0.001); 2:8(<0.001); 3:5(0.001); 3:7(<0.001); 4:6(<0.001); 4;7(<0.001); 5:6(<0.001); 5:7(<0.001); 6:8(<0.001); 7:8(<0.001) |
| Massively-Multiplayer-Online-Role-Playing-Games | 1.42±0.90 | 1.59±1.11 | 1.47±0.91 | 1.13±0.49 | 1.34±0.74 | 1.44±0.94 | 1.71±1.16 | 1.23±0.63 | **<0.001** | 2:8(<0.001); 4:7(<0.001); 5:7(<0.001); 7:8(<0.001) |
| Skill games & Time wasters | 1.99±1.21 | 2.16±1.22 | 2.32±1.21 | 1.79±1.18 | 1.72±0.98 | 1.79±1.07 | 1.95±1.24 | 1.78±1.08 | **<0.001** | 2:5(<0.001); 2:7(<0.001); 2:8(<0.001); 3:5(<0.001); 3:6(0.001); 3:7(<0.001); 3:8(<0.001) |
| Internet gaming | 1.65±1.08 | 1.49±0.90 | 1.53±0.88 | 1.28±0.74 | 1.52±0.95 | 1.51±0.91 | 1.79±1.13 | 1.29±0.68 | **<0.001** | 2:7(<0.001); 4:7(<0.001); 5:7(0.001); 7:8(<0.001) |
| Online Shopping | 2.54±1.00 | 2.32±0.99 | 2.04±0.96 | 2.21±0.81 | 2.13±0.93 | 2.91±0.99 | 2.46±1.08 | 2.50±0.99 | **<0.001** | 1:3(<0.001); 1:5(<0.001); 2:3(<0.001); 2:6(<0.001); 3:6(<0.001); 3:7(<0.001) 3:8(<0.001); 4:6(<0.001); 5:6(<0.001); 5:7(<0.001); 5:8(<0.001); 6:7(<0.001) |
| Online gambling | 1.13±0.53 | 1.05±0.34 | 1.05±0.34 | 1.19±0.61 | 1.08±0.37 | 1.10±0.39 | 1.11±0.45 | 1.04±0.23 | **0.003** | 2:7(0.001) |
| Social networking | 3.66±0.93 | 3.89±0.99 | 3.55±1.17 | 2.98±1.05 | 3.59±0.92 | 3.41±1.10 | 3.94±1.07 | 3.48±1.02 | **<0.001** | 1:4(<0.001); 1:7(<0.001); 2:3(<0.001); 2:4(<0.001); 2:5(<0.001); 2:6(<0.001); 2:8(<0.001); 3:4(<0.001); 3:7(<0.001); 4:7(<0.001); 5:7(<0.001); 6:7(<0.001); 7:8(<0.001) |

| | | | | | | | | | | |
|---|---|---|---|---|---|---|---|---|---|---|
| Sports | 1.95±1.17 | 2.47±1.34 | 2.37±1.31 | 2.49±1.33 | 2.27±1.16 | 1.79±1.17 | 2.18±1.18 | 2.17±1.21 | **<0.001** | 1:2(<0.001); 2:6(<0.001); 2:7(<0.001); 3:6(<0.001) |
| Pornography viewing | 1.79±1.11 | 2.09±1.18 | 1.54±0.86 | 1.57±0.95 | 1.46±0.87 | 1.74±1.07 | 2.06±1.18 | 1.32±0.67 | **<0.001** | 1:8(<0.001); 2:3(<0.001); 2:5(<0.001); 2:8(<0.001); 3:7(<0.001); 5:7(<0.001); 7:8(<0.001) |
| Streaming | 3.06±1.27 | 3.64±1.07 | 2.92±1.25 | 2.47±1.14 | 3.09±1.15 | 3.06±1.11 | 3.80±1.14 | 3.05±1.07 | **<0.001** | 1:2(<0.001); 1:7(<0.001); 2:3(<0.001); 2:4(<0.001); 2:5(<0.001); 2:6(<0.001); 2:8(<0.001); 3:7(<0.001); 4:7(<0.001); 5:7(<0.001); 6:7(<0.001); 7:8(<0.001) |
| Digital Hoarding | 1.70±0.97 | 1.84±1.02 | 2.31±1.11 | 1.49±0.80 | 2.08±1.12 | 1.93±1.12 | 2.40±1.26 | 1.66±0.92 | **<0.001** | 1:3(<0.001); 1:7(<0.001); 2:3(<0.001); 2:7(<0.001); 3:4(<0.001); 3:8(<0.001); 4:5(0.001); 4:7(<0.001); 5:8(<0.001); 6:7(.002); 7:8(<0.001) |
| Cyberchondria | 1.64±1.00 | 1.50±0.86 | 1.64±0.87 | 1.47±0.75 | 1.53±0.90 | 1.41±0.76 | 1.91±1.09 | 1.70±0.87 | **<0.001** | 2:7(<0.001); 2:8(<0.001); 3:7(0.001); 5:7(<0.001); 6:7(<0.001) |
| **Browsing online hours per day during periods of increased social/ physical distance** | 6.20±3.80 | 5.54±3.01 | 4.12±2.65 | 5.30±3.92 | 5.72±3.77 | 4.28±2.60 | 5.28±3.75 | 4.26±2.81 | **<0.001** | 1:3(<0.001); 1:6(<0.001); 1:8(<0.001); 2:3(<0.001); 2:6(<0.001); 2:7(<0.001); 2:8(<0.001); 3:5(<0.001); 3:7(<0.001); 5:8(<00.001); 7:8(<00.001) |

Note: * Comparison between two countries.

**Table S4.** Mean scores of appearance anxiety self-compassion measures and time spent online across countries.

| | **Lithuania** | **Hungary** | **Spain** | **Italy** | **UK** | **Portugal** | **Japan** | **Brazil** | **F** | **p** |
|---|---|---|---|---|---|---|---|---|---|---|
| AAI | 16.30±4.55 | 16.62±4.94 | 14.78±4.08 | 16.28±5.25 | 16.09±5.47 | 16.37±5.22 | 16.01±4.81 | 18.55±6.24 | **17.61** | **0.000** |
| Global SCS | 33.45±5.64 | 33.43±7.02 | 32.43±5.65 | 29.79±5.18 | 29.79±6.52 | 33.10±5.49 | 32.97±5.58 | 29.99±6.44 | **20.65** | **0.000** |
| Hours | 4.26±2.81 | 5.30±3.92 | 4.12±2.65 | 5.54±3.01 | 6.20±3.80 | 5.72±3.77 | 4.28±2.60 | 5.28±3.75 | **10.28** | **0.000** |

**Table S5.** Cross cultural comparisons of time spent online, appearance anxiety and self-compassion measures.

| Multiple Comparisons | | | | | | | | |
|---|---|---|---|---|---|---|---|---|
| **Dependent Variable** | | **(I) Country** | **(J) Country** | **Mean Difference (I-J)** | **Std. Error** | **Sig.** | **95% Confidence Interval** | |
| | | | | | | | **Lower Bound** | **Upper Bound** |
| AAI | Tahmane | Lithuania | Spain | 1.521 | 0.398 | 0.004 | 0.27 | 2.77 |
| | | | Brazil | -2.251 | 0.385 | 0.000 | -3.46 | -1.04 |
| | | Hungary | - | - | - | - | - | - |
| | | Spain | Lithuania | -1.521 | 0.398 | 0.004 | -2.77 | -0.27 |
| | | | Italy | -1.495 | 0.330 | 0.000 | -2.53 | -0.46 |
| | | | Portugal | -1.590 | 0.472 | 0.024 | -3.07 | -0.11 |
| | | | Brazil | -3.772 | 0.346 | 0.000 | -4.85 | -2.69 |
| | | Italy | Spain | 1.495 | 0.330 | 0.000 | 0.46 | 2.53 |
| | | | Brazil | -2.277 | 0.315 | 0.000 | -3.26 | -1.29 |
| | | UK | Brazil | -2.467 | 0.541 | 0.000 | -4.18 | -0.76 |
| | | Portugal | Spain | 1.590 | 0.472 | 0.024 | 0.11 | 3.07 |
| | | | Brazil | -2.182 | 0.461 | 0.000 | -3.63 | -0.73 |
| | | Japan | Brazil | -2.539 | 0.629 | 0.003 | -4.56 | -0.52 |
| | | Brazil | Lithuania | 2.251 | 0.385 | 0.000 | 1.04 | 3.46 |
| | | | Spain | 3.772 | 0.346 | 0.000 | 2.69 | 4.85 |
| | | | Italy | 2.277 | 0.315 | 0.000 | 1.29 | 3.26 |
| | | | UK | 2.467 | 0.541 | 0.000 | 0.76 | 4.18 |
| | | | Portugal | 2.182 | 0.461 | 0.000 | 0.73 | 3.63 |
| | | | Japan | 2.539 | 0.629 | 0.003 | 0.52 | 4.56 |
| SCS | Tahmane | Lithuania | Italy | 3.663 | 0.432 | 0.000 | 2.31 | 5.02 |
| | | | UK | 3.659 | 0.694 | 0.000 | 1.47 | 5.85 |
| | | | Brazil | 3.461 | 0.450 | 0.000 | 2.05 | 4.87 |
| | | Hungary | Italy | 3.636 | 1.045 | 0.029 | 0.20 | 7.08 |
| | | Spain | Italy | 2.639 | 0.409 | 0.000 | 1.36 | 3.92 |
| | | | UK | 2.634 | 0.680 | 0.004 | 0.49 | 4.78 |
| | | | Brazil | 2.436 | 0.427 | 0.000 | 1.10 | 3.78 |
| | | Italy | Lithuania | -3.663 | 0.432 | 0.000 | -5.02 | -2.31 |
| | | | Hungary | -3.636 | 1.045 | 0.029 | -7.08 | -0.20 |
| | | | Spain | -2.639 | 0.409 | 0.000 | -3.92 | -1.36 |
| | | | Portugal | -3.310 | 0.467 | 0.000 | -4.78 | -1.84 |
| | | | Japan | -3.182 | 0.707 | 0.001 | -5.46 | -0.90 |
| | | UK | Lithuania | -3.659 | 0.694 | 0.000 | -5.85 | -1.47 |
| | | | Spain | -2.634 | 0.680 | 0.004 | -4.78 | -0.49 |
| | | | Portugal | -3.305 | 0.716 | 0.000 | -5.56 | -1.05 |
| | | | Japan | -3.177 | 0.892 | 0.014 | -6.00 | -0.35 |
| | | Portugal | Italy | 3.310 | 0.467 | 0.000 | 1.84 | 4.78 |
| | | | UK | 3.305 | 0.716 | 0.000 | 1.05 | 5.56 |

| | | | | | | | | |
|---|---|---|---|---|---|---|---|---|
| | | | Brazil | 3.107 | 0.483 | 0.000 | 1.59 | 4.63 |
| | | Japan | Italy | 3.182 | 0.707 | 0.001 | 0.90 | 5.46 |
| | | | UK | 3.177 | 0.892 | 0.014 | 0.35 | 6.00 |
| | | | Brazil | 2.979 | 0.718 | 0.002 | 0.67 | 5.29 |
| | | Brazil | Lithuania | -3.461 | 0.450 | 0.000 | -4.87 | -2.05 |
| | | | Spain | -2.436 | 0.427 | 0.000 | -3.78 | -1.10 |
| | | | Portugal | -3.107 | 0.483 | 0.000 | -4.63 | -1.59 |
| | | | Japan | -2.979 | 0.718 | 0.002 | -5.29 | -0.67 |
| Hours | Tahmane | Lithuania | Italy | -1.27967 | 0.22439 | 0.000 | -1.9835 | -0.5758 |
| | | | UK | -1.93488 | 0.38788 | 0.000 | -3.1598 | -0.7100 |
| | | | Portugal | -1.46057 | 0.34424 | 0.001 | -2.5428 | -0.3783 |
| | | | Brazil | -1.02233 | 0.23566 | 0.000 | -1.7608 | -0.2838 |
| | | Hungary | - | - | - | - | - | - |
| | | Spain | Italy | -1.41641 | 0.20485 | 0.000 | -2.0580 | -0.7748 |
| | | | UK | -2.07162 | 0.37691 | 0.000 | -3.2633 | -0.8799 |
| | | | Portugal | -1.59731 | 0.33183 | 0.000 | -2.6414 | -0.5533 |
| | | | Brazil | -1.15907 | 0.21713 | 0.000 | -1.8386 | -0.4795 |
| | | Italy | Lithuania | 1.27967 | 0.22439 | 0.000 | 0.5758 | 1.9835 |
| | | | Spain | 1.41641 | 0.20485 | 0.000 | 0.7748 | 2.0580 |
| | | | Japan | 1.26062 | 0.33711 | 0.009 | 0.1772 | 2.3441 |
| | | UK | Lithuania | 1.93488 | 0.38788 | 0.000 | 0.7100 | 3.1598 |
| | | | Spain | 2.07162 | 0.37691 | 0.000 | 0.8799 | 3.2633 |
| | | | Japan | 1.91583 | 0.46232 | 0.001 | 0.4535 | 3.3782 |
| | | Portugal | Lithuania | 1.46057 | 0.34424 | 0.001 | 0.3783 | 2.5428 |
| | | | Spain | 1.59731 | 0.33183 | 0.000 | 0.5533 | 2.6414 |
| | | | Japan | 1.44152 | 0.42637 | 0.025 | 0.0924 | 2.7906 |
| | | Japan | Italy | -1.26062 | 0.33711 | 0.009 | -2.3441 | -0.1772 |
| | | | UK | -1.91583 | 0.46232 | 0.001 | -3.3782 | -0.4535 |
| | | | Portugal | -1.44152 | 0.42637 | 0.025 | -2.7906 | -0.0924 |
| | | Brazil | Lithuania | 1.02233 | 0.23566 | 0.000 | 0.2838 | 1.7608 |
| | | | Spain | 1.15907 | 0.21713 | 0.000 | 0.4795 | 1.8386 |

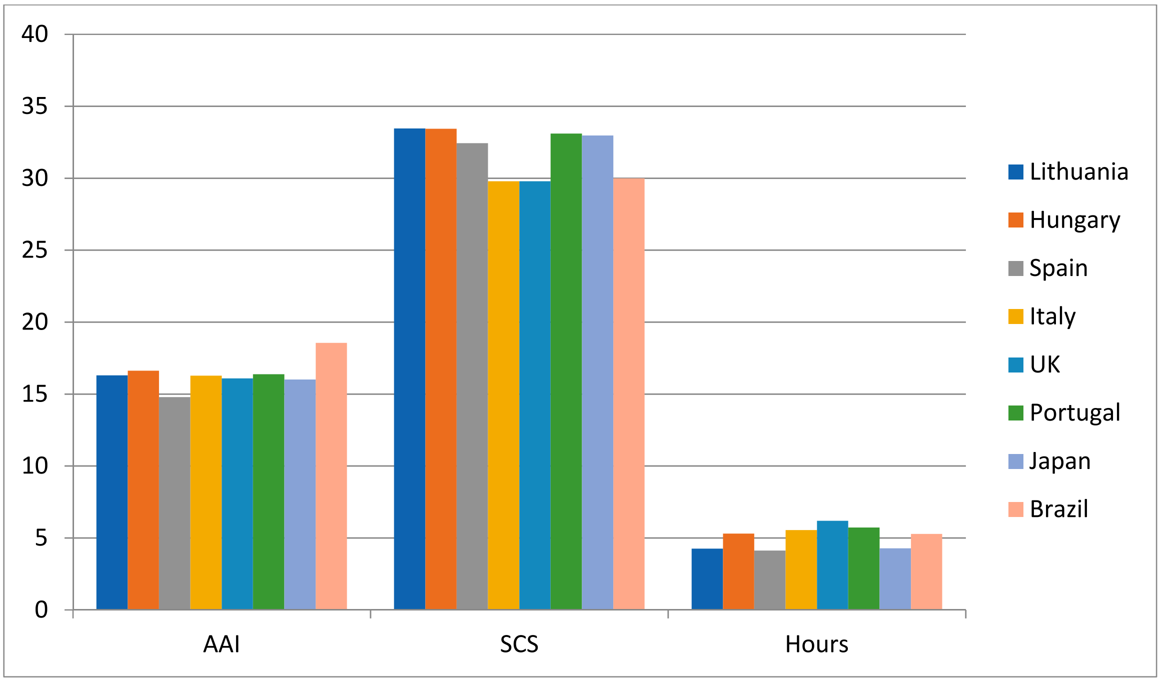


**Figure S1.** Appearance anxiety, self-compassion measures and time spent online in different countries.
